# Comprehensive Genomic Profiling of Advanced Anal Adenocarcinoma in Japan

**DOI:** 10.1200/PO-25-00562

**Published:** 2026-01-15

**Authors:** Nozomu Ogura, Hidekazu Hirano, Kouya Shiraishi, Hiroyuki Fujii, Toshiharu Hirose, Hirokazu Shoji, Natsuko Okita, Atsuo Takashima, Takafumi Koyama, Kan Yonemori, Ken Kato

**Affiliations:** ^1^ Department of Gastrointestinal Medical Oncology, National Cancer Center Hospital, Tokyo, Japan; ^2^ Jikei University School of Medicine, Tokyo, Japan; ^3^ Division of Genome Biology, National Cancer Center Research Institute, Tokyo, Japan; ^4^ Department of Clinical Genomics, National Cancer Center Research Institute, Tokyo, Japan; ^5^ Department of Pulmonary Medicine, Graduate School of Medical Science, Kyoto Prefectural University of Medicine, Kyoto, Japan; ^6^ Department of Experimental Therapeutics, National Cancer Center Hospital, Tokyo, Japan; ^7^ Department of Medical Oncology, National Cancer Center Hospital, Tokyo, Japan

## Abstract

**PURPOSE:**

Anal adenocarcinoma (AD) is a rare GI malignancy with no established standard treatment. Little is known about genomic alterations (GAs) and their therapeutic implications in advanced anal AD. Here, we compared the genomic profiles of advanced anal AD and advanced rectal AD.

**METHODS:**

We retrospectively extracted data from patients with advanced anal or rectal AD who underwent comprehensive genomic profiling (CGP) and were registered at the Center for Cancer Genomics and Advanced Therapeutics (C-CAT) in Japan. We examined somatic GAs, microsatellite instability (MSI) status, and tumor mutation burden (TMB).

**RESULTS:**

From June 2019 to April 2023, 45 patients with anal AD and 1,915 patients with rectal AD were enrolled into the C-CAT database. *TP53* (88.9%) and *KRAS* (51.1%) were the most common GAs in anal AD. Compared with rectal AD, anal AD showed significantly higher frequencies of *ERBB3* (22.2% *v* 1.8%), *MYC* (20.0% *v* 8.4%), and *BRCA2* (6.7% *v* 1.5%) alterations and a significantly lower frequency of *APC* mutations (8.9% *v* 84.6%). TMB-high status (≥10 mutations/Mb) was observed in 6.7% of anal AD cases, whereas no MSI-high tumors were identified in this group. At least one druggable GA (excluding *RAS*, *BRAF* V600E, *ERBB2*, and MSI) was detected in 40.0% of patients with anal AD. Druggable GAs were identified in genes related to the MAPK pathway, DNA damage response pathway, and other oncogenic pathways.

**CONCLUSION:**

Advanced anal AD exhibited a distinct genomic profile compared with advanced rectal AD. CGP is a useful approach for identifying druggable GAs in advanced anal AD to expand therapeutic opportunities.

## INTRODUCTION

Anal canal cancer is a rare tumor that accounts for 2.5% of GI malignancies.^1^ According to the TNM Classification of Malignant Tumors (8th edition) of the Union for International Cancer Control, anal canal cancer is defined as a tumor arising from the surgical anal canal, extending from the anorectal ring to the anal verge or the perianal skin within 5 cm from the anal verge.^2^ Histologically, this cancer includes squamous cell carcinoma, adenocarcinoma (AD), lymphoma, GI stromal tumor, melanoma, and neuroendocrine tumor^3^; squamous cell carcinoma is the most common worldwide, whereas AD is relatively rare at approximately 20% of cases.^4^

**CONTEXT**

**Key Objective**
What genomic alterations (GAs) characterize advanced anal adenocarcinoma (AD) based on real-world comprehensive genomic profiling (CGP)?
**Knowledge Generated**
Compared with rectal AD (n = 1,915), anal AD (n = 45) exhibits higher frequencies of actionable mutations in ERBB3, MYC, and BRCA2, while APC was significantly lower. In the anal AD, druggable GAs with approved drugs were identified in 11.1%, including olaparib and niraparib for BRCA2, pemigatinib for FGFR2, and pembrolizumab for tumor mutation burden-high.
**Relevance**
These findings underscore the potential of personalized treatment strategies in advanced anal AD and highlight the role of CGP in guiding therapeutic decisions. Future clinical development may make novel therapeutic agents available for advanced anal AD harboring actionable GAs.

Because of its rarity, there is a lack of sufficient studies to create guidelines recommending uniform treatment methods for advanced anal AD and the treatment strategies used for advanced rectal AD are generally extrapolated. In unresectable advanced anal AD, the choice of systematic therapy is determined according to its molecular characteristics. *RAS*, *BRAF*, *ERBB2*, and microsatellite instability (MSI)/mismatch repair testing for rectal AD are performed widely to guide the use of anti-epidermal growth factor receptor (EGFR) antibodies, BRAF inhibitors, anti–human epidermal growth factor receptor 2 (HER2) antibodies, and immune checkpoint inhibitors, which have shown efficacy in patients with each specific type of tumor.^5-8^

Furthermore, comprehensive genomic profiling (CGP) by next-generation sequencing (NGS) was approved by the US Food and Drug Administration (FDA) in 2017 and is currently used in routine clinical practice for patients with various solid tumors.^9^ Comprehensive analysis of genomic alterations (GAs) in tumors by CGP has led to therapeutic approaches with molecular-targeted therapies, enabling precision medicine. In rectal cancer, GAs have been identified by CGP, and treatments targeting these GAs are available.^10^ However, little is known about GAs and their therapeutic implications in advanced anal AD, and its genomic profile remains unclear. In addition, there is a scarcity of data on GAs in advanced anal AD compared with rectal AD.

Therefore, we investigated the status of GAs of patients with advanced anal AD in comparison with those with advanced rectal AD by CGP and evaluated the frequency of GAs that could be therapeutic targets.

## METHODS

### Study Population and Data Collection

Data were extracted retrospectively for patients with advanced anal or rectal AD who underwent CGP and were registered at the Center for Cancer Genomics and Advanced Therapeutics (C-CAT) in Japan on May 2, 2023 (ver.6.00). The C-CAT database comprehensively aggregates clinical and genomic information on Japanese patients with an advanced malignant tumor who underwent CGP by NGS.^11^ All these patients had completed or were nearing completion of standard treatment for their tumor. Two different platforms were used for NGS: OncoGuide NCC Oncopanel System^12^ and FoundationOne CDx Cancer Genomic Profile.^13^

Clinical and genomic data were obtained from the C-CAT database, and the period of data was from June 2019 to April 2023. The clinical data included age, sex, Eastern Cooperative Oncology Group Performance Status (ECOG-PS), sample collection timing and method (surgery or biopsy), and CGP platform. Clinical characteristics such as age and ECOG-PS were those at the time of CGP. Genomic data included GAs, their variation types, annotated clinical significance by C-CAT, MSI status, tumor mutation burden (TMB), and information on drugs expected to be effective against those GAs. Small-scale variants (SSVs) included single-nucleotide variants, insertions, and deletions. Amplification is defined as a copy number ratio of 4 or more in the NCC Oncopanel, whereas in FoundationOne CDx, the thresholds are four for *ERBB2* and six for all other genes.^12,13^ Multiple class alterations indicate the simultaneous combination of SSVs and other types of variation in the same gene. In this study, we exclusively examined somatic GAs. TMB-high was defined as a TMB of ≥10 mutations per megabase (Muts/Mb). The Institutional Review Board of the National Cancer Center Hospital provided ethical approval for this study (approval No. 2020-067), followed by approval from the C-CAT Data Utilization Review Board (approval No. CDU2021-001N). All procedures were conducted in accordance with the Declaration of Helsinki.

### Annotation and Extraction of Candidate Drugs

CGP results were annotated by a curator team of medical oncologists using C-CAT proprietary algorithms,^11^ Association of Molecular Pathology, ASCO, and College of American Pathologists joint guidelines^14^ and public databases (COSMIC,^15^ ClinVar,^16^ CIViC,^17^ and BRCA Exchange^18^). In this study, GAs classified as “pathogenic,” “oncogenic,” “likely pathogenic,” and “likely oncogenic” variants were defined as actionable GAs associated with disease progression in anal and rectal AD pathogenesis. Variants of unknown significance were not included as actionable GAs.

The extraction of candidate drugs targeting actionable GAs and the interpretation of the evidence level were evaluated using C-CAT evidence levels of anticancer agents that are approved/underdevelopment in Japan or the FDA and accompanied by the corresponding biomarkers (Appendix Table A1).^11^ In this study, druggable GAs were defined as actionable GAs with approved drugs for anal AD, rectal AD, or other types of cancers or with clinical trials showing efficacy for either anal and rectal AD or different types of cancers, for which a specific drug was recommended at C-CAT evidence level C or higher. The extraction of druggable GA data was performed as of May 2023.

### Statistical Analysis

Descriptive statistics were used to summarize the baseline characteristics of the patients. Fisher's exact test or the chi-square test for categorical data and Student's *t*-test for continuous data were used to compare the characteristics between the two groups. Statistical tests were two-sided, and a *P* value of <.05 was considered statistically significant. Overall survival (OS) was defined as the time from the beginning of first-line systemic chemotherapy to death from any cause or censoring at the latest follow-up for surviving patients. Probabilities of survival were estimated using the Kaplan-Meier method and compared using the log-rank test. Hazard ratios (HRs) were calculated using the Cox proportional hazards model. All statistical analyses were performed using the R statistical software package (The R Foundation for Statistical Computing, Vienna, Austria).

## RESULTS

### Patient Characteristics

Forty-five patients with advanced anal AD and 1,915 patients with rectal AD were enrolled into the C-CAT database. Their baseline characteristics are summarized in Table 1. The median age was 67 years (range, 36-79 years) and 61 years (range, 20-86 years), 23 (51.1%) and 1,173 (61.3%) were male in the anal and rectal AD groups, respectively, and almost all patients had an ECOG-PS of 0 or 1. The NCC Oncopanel was used in five (11.1%) and 153 (8.0%) cases, and FoundationOne CDx was used in 40 (88.9%) and 1,762 (92.0%) cases in the anal and rectal AD groups, respectively. In both groups, surgical specimens were used most frequently as samples for NGS. There were no significant differences between the anal and rectal AD groups regarding age, sex, ECOG-PS, sample collection method, and CGP platform.

**TABLE 1.** Patient Characteristics

Characteristic	Anal AD (n *=* 45)	Rectal AD (n *=* 1,915)	*P*
Age, years, median (range)	67 (36-79)	61 (20-86)	.11
Sex, No. (%)			.17
Male	23 (51.1)	1,173 (61.3)	
Female	22 (48.9)	742 (38.7)	
ECOG-PS, No. (%)			.69
0	32 (71.1)	1,179 (61.6)	
1	11 (24.4)	557 (29.1)	
≥2	0 (0)	43 (2.2)	
Not available	2 (4.4)	136 (7.1)	
Metastatic organ, No. (%)			
Liver	14 (31.1)	996 (52.0)	.006
Lung/pleura	20 (44.4)	1,088 (56.8)	.10
Lymph node	22 (48.9)	624 (32.6)	.02
Peritoneum	5 (11.1)	325 (17.0)	.30
Bone	10 (22.2)	133 (6.9)	<.001
Sample collection method, No. (%)			.28
Surgery	32 (71.1)	1,493 (78.0)	
Biopsy	13 (28.9)	422 (22.0)	
Sample collection site, No. (%)			.24
Primary site	27 (60.0)	1,308 (68.3)	
Metastatic site	18 (40.0)	607 (31.7)	
Sample collection timing, No. (%)			.39
Pretreatment	19 (42.2)	977 (51.0)	
Post-treatment	21 (46.7)	705 (36.8)	
Unknown	5 (11.1)	233 (12.2)	
CGP platform, No. (%)			.45
NCC Oncopanel	5 (11.1)	153 (8.0)	
FoundationOne CDx	40 (88.9)	1,762 (92.0)	

Abbreviations: AD, adenocarcinoma; CGP, comprehensive genomic profiling; ECOG-PS, Eastern Cooperative Oncology Group Performance Status.

### Actionable GAs, MSI, and TMB

**FIG 1.**
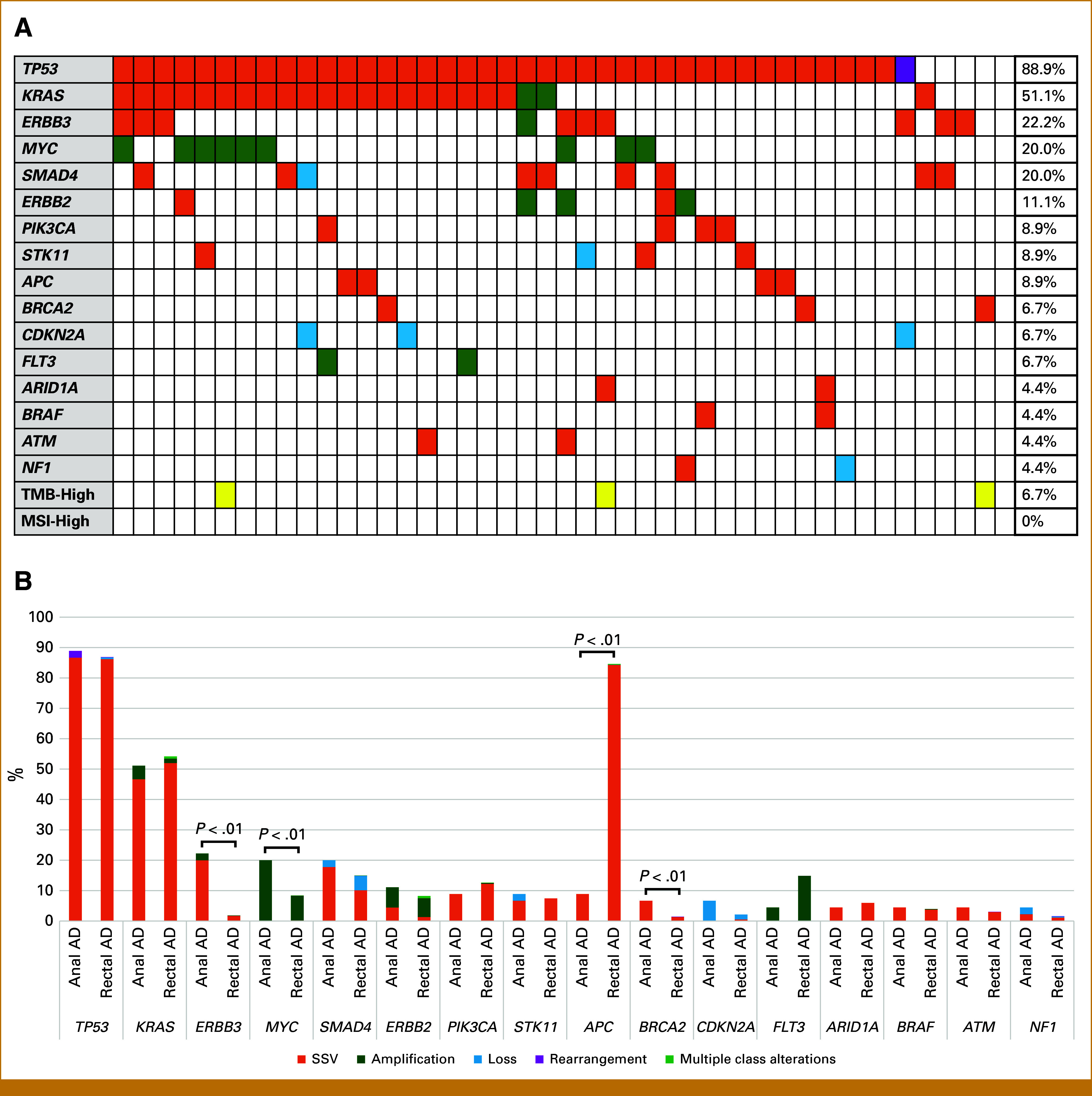
(A) Landscape in advanced anal AD and (B) bar graph of actionable genomic alterations in specific genes detected at a frequency of >3% and TMB-high and MSI-high status. AD, adenocarcinoma; MSI, microsatellite instability; SSV, small-scale variant; TMB, tumor mutation burden.

The actionable GAs detected at a frequency of >3% in the anal AD group and TMB-high and MSI-high status are shown in Figures 1A and 1B. In the anal AD group, the most common actionable GAs were *TP53* (88.9% [40 of 45]), *KRAS* (51.1% [23 of 45]), *ERBB3* (22.2% [10 of 45]), *MYC* (20.0% [9 of 45]), *SMAD4* (20.0% [9 of 45]), *ERBB2* (11.1% [5 of 45]), *PIK3CA* (8.9% [4 of 45]), *STK11* (8.9% [4 of 45]), and *APC* (8.9% [4 of 45]; Appendix Table A2). Compared with rectal AD, anal AD had a significantly higher frequency of actionable GAs in *ERBB3* (22.2% *v* 1.8%, *P* < .01), *MYC* (20.0% *v* 8.4%, *P* < .01), and *BRCA2* (6.7% *v* 1.5%, *P* < .01) and had a significantly lower frequency of actionable GAs in *APC* (8.8% *v* 84.6%, *P* < .01). There was a trend toward lower frequency of *FLT3* amplification in anal AD (4.4% *v* 14.7%, *P* = .0528). Among the anal AD group, the most common mutations of *ERBB3* were G284R (11.1% [5 of 45], extracellular domain II), V104L (4.4% [2 of 45], extracellular domain I), and A232V/R1118Q (2.2% [1 of 45], extracellular domain II/C-terminal tail; Appendix Table A3).^19^

*RAS*, *BRAF* V600E, *ERBB2*, and MSI status, markers commonly used for selecting treatment in clinical practice, are shown in Table 2. The frequency of anal AD with *RAS*-mutated, *BRAF* V600E–mutated, and *ERBB2*-amplified was 48.8%, 2.2%, and 6.7%, respectively. No patients had MSI-high status in the anal AD group. There were no significant differences in the frequencies of *RAS*-mutated, *BRAF* V600E–mutated, *ERBB2*-amplified, or MSI-high tumors between the anal and rectal AD groups.

**TABLE 2.** Status of *RAS*, *BRAF* V600E, *ERBB2*, and MSI

Biomarker Status	Anal AD (n *=* 45), No. (%)	Rectal AD (n *=* 1,915), No. (%)	*P*
*RAS*			.93
Wild-type	22 (48.9)	924 (48.3)	
Mutated			
*KRAS* G12C	0 (0)	52 (2.7)	
*KRAS* G12D	10 (22.2)	314 (16.4)	
Non-*KRAS* G12C/G12D	13 (28.9)	625 (32.6)	
*BRAF* V600E			.87
Wild-type	44 (97.8)	1,879 (98.1)	
Mutated	1 (2.2)	36 (1.9)	
*ERBB2*			.95
Not amplified	42 (93.3)	1,783 (93.1)	
Amplified	3 (6.7)	132 (6.9)	
MSI-high			.79
Not detected	45 (100)	1,912 (99.8)	
Detected	0 (0)	3 (0.2)	

Abbreviations: AD, adenocarcinoma; MSI, microsatellite instability.

**FIG 2.**
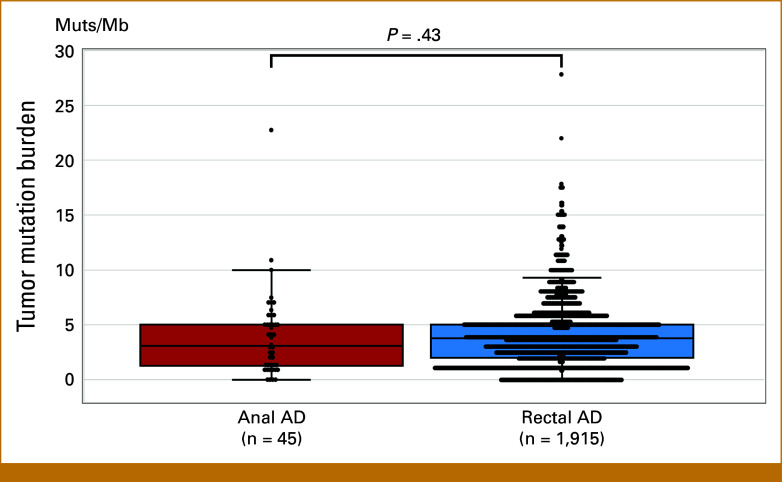
Distribution of the tumor mutation burden. AD, adenocarcinoma; Muts/Mb, mutations per megabase.

TMB-high status was observed in 6.7% (3 of 45) of patients with anal AD and 4.8% (92 of 1,915) of patients with rectal AD (Fig 2). There was no significant difference in the TMB between the anal and rectal AD groups (*P* = .43). In the three TMB-high anal AD cases, the TMB values were 10.0, 11.0, and 22.7 Muts/Mb; all were measured with FoundationOne CDx. Median TMB values were 4.5 and 2.39 Muts/Mb in the anal and rectal AD groups, respectively.

### Druggable GAs

**FIG 3.**
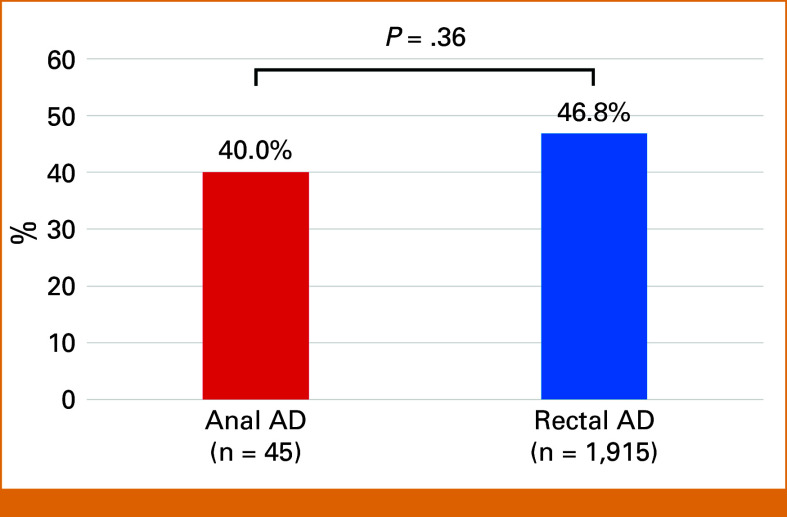
Druggable genomic alterations excluding *RAS*, *BRAF* V600E, *ERBB2* amplification, and microsatellite instability. AD, adenocarcinoma.

On the basis of the C-CAT algorithm, at least one druggable GA, excluding *RAS*-mutated, *BRAF* V600E–mutated, *ERBB2*-amplified, and MSI-high, was found in 40.0% (18 of 45) of patients with anal AD and 46.8% (897 of 1,915) of patients with rectal AD (Fig 3). Candidate drugs for each druggable GA in the C-CAT database included olaparib for SSVs of *ATM*, *BRCA2*, and *PALB2*, palbociclib for SSVs of *CDK4*, infigratinib for *FGFR1* amplification and *FGFR2* rearrangement, gilteritinib for *FLT3* amplification, imatinib for *KIT* and *PDGFRA* amplification, alpelisib for SSVs of *PIK3CA*, and pembrolizumab for TMB-high status (Table 3). There were no significant differences in the frequencies of druggable GAs between the anal and rectal AD groups (*P* = .36).

**TABLE 3.** Combination of Specific Druggable GAs and Candidate Therapeutic Drugs in Anal AD

Gene	GA Category	Candidate Drug	Drug Status	Anal AD (n *=* 45)
*PIK3CA*	SSV	Alpelisib/everolimus	Off-label use	4 (8.8)
*BRCA2*	SSV	Olaparib/niraparib/rucaparib	Off-label use	3 (6.7)
*ATM*	SSV	Olaparib	Off-label use	2 (4.4)
*FLT3*	Amplification	Gilteritinib	Off-label use	2 (4.4)
*CDK4*	Amplification	Palbociclib	Off-label use	1 (2.2)
*FGFR1*	Amplification	Infigratinib	Off-label use	1 (2.2)
*FGFR2*	Rearrangement	Infigratinib/pemigatinib	Off-label use	1 (2.2)
*KIT*	Amplification	Imatinib	Off-label use	1 (2.2)
*PALB2*	SSV	Olaparib	Off-label use	1 (2.2)
*PDGFRA*	Amplification	Imatinib	Off-label use	1 (2.2)
*PTEN*	Loss	Everolimus	Off-label use	1 (2.2)
TMB-high	Other	Pembrolizumab	Approved	3 (6.7)

Abbreviations: AD, adenocarcinoma; GA, genomic alteration; SSV, small-scale variant; TMB, tumor mutation burden.

In addition, we investigated druggable GAs with approved drugs for cancer harboring–specific GAs in Japan. In the anal AD group, 11.1% (5 of 45) of patients had druggable GAs with approved drugs, such as olaparib and niraparib for *BRCA2*, pemigatinib for *FGFR2*, and pembrolizumab for TMB-high status.

### Survival Data

**FIG A1.**
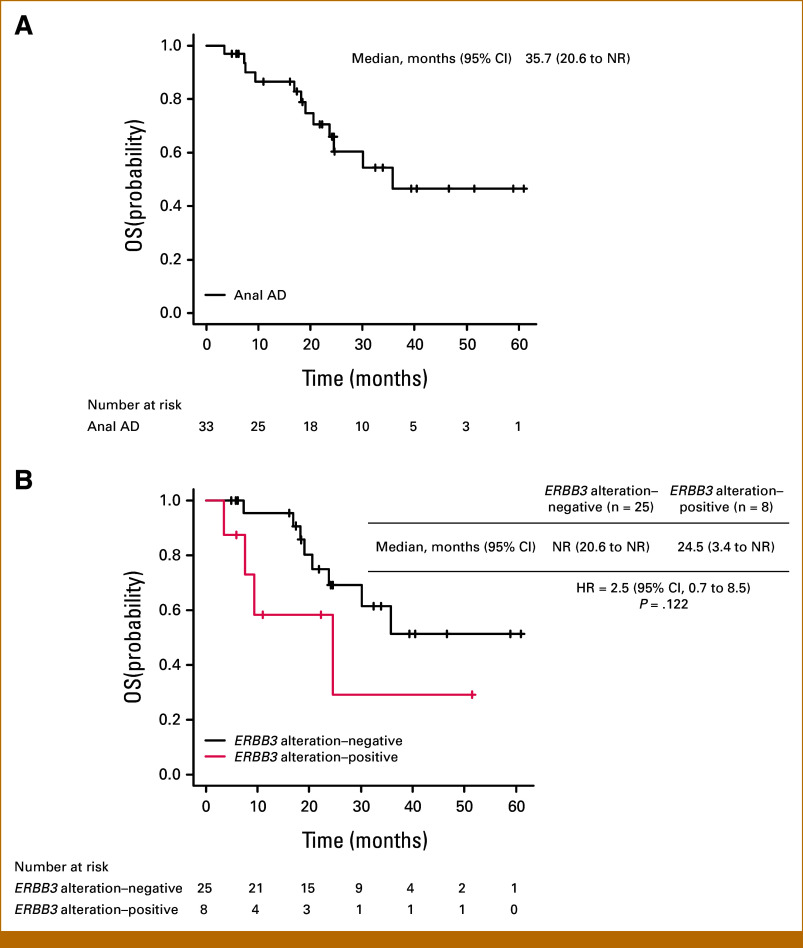
(A) OS of the anal AD group. (B) OS of the anal AD group according to *ERBB3* alteration. AD, adenocarcinoma; HR, hazard ratio; NR, not reached; OS, overall survival.

Survival data were available for 33 of 45 (73.3%) patients in the anal AD group, with a median OS of 35.7 months (95% CI, 20.6 to not reached; Appendix Fig A1A). There was a trend toward poorer OS in the *ERBB3* alteration–positive patients compared with the *ERBB3* alteration–negative patients (median OS, 24.5 months [95% CI, 3.4 to not reached] *v* not reached [95% CI, 20.6 to not reached], HR, 2.5 [95% CI, 0.7 to 8.5]; *P* = .12; Appendix Fig A1B).

## DISCUSSION

In this study, to our knowledge, we conducted the largest comparative genomic analysis of advanced anal and rectal AD using a national database of CGP results in Japan. Our findings revealed that advanced anal AD has a distinct genomic landscape compared with advanced rectal AD, for example, a low frequency of *APC* mutations, suggesting two neighboring malignancies displaying different biological features. In addition, our genomic analysis identified multiple potential targetable GAs in advanced anal AD.

We demonstrated that the most common actionable GAs in advanced anal AD were *TP53* (88.9%), *KRAS* (51.1%), *ERBB3* (22.2%), *MYC* (20.0%), and *SMAD4* (20.0%). Somatic mutations in both *TP53* and *KRAS* are the most frequent oncogenic alterations in human cancers, and defects in their function cause tumor development and growth in various types of cancers.^20,21^ In this study, the frequency of *TP53* and *KRAS* mutations was comparable between advanced anal and rectal AD, suggesting that both are mainly involved in the oncogenesis of both. Notably, anal AD was associated with a lower frequency of *APC* mutations than rectal AD (8.8% *v* 84.6%, *P* < .01). *APC* mutations activate the Wnt signaling pathway and deregulate multiple other cellular processes, which are key and early steps in the development of colorectal cancers.^22^ However, the present study indicated that this oncogenic pathway induced by *APC* mutations is not the primary mechanism in advanced anal AD.

We also found that advanced anal AD was associated with a significantly higher frequency of actionable GAs in *ERBB3* (22.2% *v* 1.8%, *P* < .01), *MYC* (20.0% *v* 8.4%, *P* < .01), and *BRCA2* (6.7% *v* 1.5%, *P* < .01) than advanced rectal AD. HER3, encoded by the *ERBB3* gene, plays a crucial role in the transformation and proliferation of many types of cancers.^23^ The prevalence of *ERBB3* somatic mutation has been reported to be 1%-6% in solid tumors,^24^ and we found that advanced anal AD had a high frequency of *ERBB3* mutations. Molecular targeted agents that inhibit aberrant signaling driven by *ERBB3* mutations are expected to provide clinical benefits; however, no effective agents are currently available, as demonstrated in the SUMMIT trial, which reported no objective responses to neratinib (a pan-HER tyrosine kinase inhibitor) in patients with tumors harboring *ERBB3* mutations.^25^ On the other hand, there are preclinical and clinical data suggesting that targeting HER2-HER3 signaling or HER3 itself may be effective against tumors harboring *ERBB3* mutations.^26-29^ Such targeted therapies might be promising for advanced anal AD. Furthermore, HER3 is associated with resistance to therapies targeting other HER receptors and chemotherapy^26^ and the higher frequency of *ERBB3* alterations in patients with advanced anal AD may support previous reports of a worse prognosis in anal AD compared with rectal AD.^30,31^ In fact, in the current study, OS tended to be worse in advanced anal AD patients with *ERBB3* alterations than in those without *ERBB3* alterations and the presence of an *ERBB3* alteration might be a prognostic factor for survival outcome in patients with advanced anal AD. MYC overexpression mainly caused by *MYC* amplification has been causally involved in the growth, progression, and maintenance of cancers of diverse origins.^32,33^ In *RAS/BRAF* wild-type metastatic colorectal cancer, high MYC expression was reported to be associated with reduced benefit from anti-EGFR antibody therapy.^34^ Mechanistically, a preclinical study indicated that forkhead transcription factors of O class 3a (FoxO3a), a regulator of cell survival, confer cetuximab resistance to *RAS* wild-type metastatic colorectal cancer via MYC.^35^ Given that *MYC* amplification was more frequent in anal AD in the present study, performing NGS and assessing *MYC* amplification may help predict resistance to anti-EGFR antibody therapy when selecting treatment for advanced anal AD.

*BRCA2*-encoded proteins are primarily involved in facilitating the homologous recombination repair of DNA damage, and defects in their function lead to dysfunctional chromosomal rearrangements and cellular replication and ultimately cancer development.^36^ Poly (ADP-ribose) polymerase (PARP) inhibitors are effective agents for *BRCA2* mutation–positive cancers, and several PARP inhibitors have been approved by the FDA for multiple types of cancers, including olaparib, rucaparib, niraparib, and talazoparib.^37^ These distinct genetic features have potential implications for oncogenesis and progression of anal AD. In fact, a previous study showed that anal AD exhibits different characteristics compared with rectal AD,^31^ for example, anal AD is pathologically dominated by high-grade histologic types such as mucinous AD and poorly differentiated AD, and the frequency of inguinal lymph node metastases is higher than that of rectal AD.^31^ In addition, unlike rectal cancer, human papillomavirus (HPV) infection is detected in approximately 50% of anal gland or transitional type AD^38^ and HPV infection may be one of the major mechanisms for the development of advanced anal AD as with anal squamous cell carcinoma. HPV-positive anal AD had a higher frequency of *PIK3CA* mutations and lower frequencies of *KRAS* and *NRAS* mutations compared with HPV-negative anal AD, supporting the existence of biologically distinct subtypes according to HPV infection status.^39^

Several guidelines recommend the exploration of the treatment landscape for advanced rectal and anal AD.^3,40^ Biomarker information such as *RAS*/*BRAF* V600E mutation, *ERBB2* amplification, MSI, and TMB is the foundation for determining the appropriate molecular-targeted drugs. The frequencies of *RAS*-mutated (51.1% *v* 51.749%, *P* = .93), *BRAF* V600E–mutated (2.2% *v* 1.9%, *P* = .87), *ERBB2*-amplified (6.7% *v* 6.9%, *P* = .95), and MSI-high (0% *v* 0.2%, *P* = .79) tumors were comparable between the advanced anal and rectal AD groups. Encorafenib plus cetuximab therapy for *BRAF* V600E–mutated, pertuzumab plus trastuzumab therapy for *ERBB2*-amplified, and pembrolizumab monotherapy, nivolumab monotherapy, and nivolumab plus ipilimumab therapy for MSI-high tumors have been shown to be effective and approved by the FDA for advanced colorectal cancer.^6,8,41,42^ The frequency of TMB-high was also comparable between the advanced anal and rectal AD groups. However, reports of treatment outcomes by these rectal AD-based treatments for patients with advanced anal AD are scarce, and it is not clear whether they are as effective in advanced anal AD as in colorectal cancer. More studies are awaited to verify whether rectal AD-based treatments are as effective in anal AD as in colorectal cancer.

Given the rarity of anal AD and the lack of established disease-specific therapeutic strategies, the identification of actionable GAs in approximately 40% of cases—excluding *RAS* and *BRAF* V600E mutations, *ERBB2* amplification, and MSI—represents a significant opportunity to expand therapeutic avenues for this malignancy. Druggable GAs included genes related to the mTOR pathway (*PIK3CA* [8.8%] and *PTEN* [2.2%]), DNA damage response pathway (*BRCA2* [6.7%], *ATM* [4.4%], and *PALB2* [2.2%]), and other oncogenic pathways (*FLT3* [4.4%], *FGFR1* [2.2%], *FGFR2* [2.2%], and *CDK4* [2.2%]). As summarized in Table 3, drugs targeting the respective GAs might be suggested as potential treatment options. In this study, 6.7% of anal AD was classified as TMB-high tumor, indicating that immune checkpoint inhibitors, including pembrolizumab, may represent a viable therapeutic option. In recent years, the development of precision medicine based on GAs has been actively pursued. On the basis of current and further clinical development, novel therapeutic agents may become available for tumors harboring gene alterations such as *KRAS* mutations (eg, LY4066434 [ClinicalTrials.gov identifier:NCT06607185]), *ERBB3* mutations (eg, HMBD-001 [ClinicalTrials.gov identifier: NCT05919537]), and *MYC* amplification (eg, KB-0742 [ClinicalTrials.gov identifier: NCT04718675]). Given the high frequency of these alterations in advanced anal AD, the expansion of clinical development in other malignancies for the assessment of their clinical efficacy in advanced anal AD is warranted.

Our study has certain limitations that should be noted. First, although it is sometimes difficult to distinguish between advanced anal and rectal AD because of their tumor spread pattern, diagnosis was performed at each institution and may not be reliable. Second, the annotation and extraction of candidate drugs were based solely on the C-CAT algorithm and were not validated through a molecular tumor board. Finally, we did not evaluate the efficacy of genome-matched therapy, in which druggable GAs were found and targeted, because of the limited availability of the recommended treatments and insufficient efficacy and survival data.

In conclusion, we determined the genomic profiles of patients with advanced anal or rectal AD by using a large-scale CGP database. Comparison with advanced rectal AD revealed various genomic features of advanced anal AD, and CGP may be a useful tool for identifying druggable GAs to expand therapeutic opportunities in advanced anal AD. As the development of precision medicine continues to advance dramatically, we hope that our results provide some insights into the treatment of advanced anal AD.

## APPENDIX

**TABLE A1.** C-CAT Evidence Levels

Evidence Level	Criteria
A	Biomarkers that predict response to PMDA- or FDA-approved therapies for a specific type of tumor Biomarkers included in professional guidelines that predict response to therapies for a specific type of tumor
B	Biomarkers that predict response to therapies for a specific type of tumor based on well-powered studies with consensus from experts in the field
C	Biomarkers that predict response to therapies approved by the PMDA or FDA for another type of tumor Biomarkers that predict response to therapies for another type of tumor based on clinical studies
D	Biomarkers that predict response to therapies for any type of tumor based on case reports
E	Biomarkers that show plausible therapeutic significance based on preclinical studies
F	Biomarkers that are associated with tumorigenesis

Abbreviations: C-CAT, Center for Cancer Genomics and Advanced Therapeutics; FDA, US Food and Drug Administration; PMDA, Pharmaceuticals and Medical Devices Agency.

**TABLE A2.** *KRAS* and *BRAF* Mutation Types

Genomic Alterations	Anal AD (n *=* 45), No. (%)	Rectal AD (n *=* 1,915), No. (%)
*RAS*		
Wild-type	22 (48.9)	924 (48.3)
Mutated		
*KRAS* G12D	10 (22.2)	314 (16.4)
*KRAS* G12V	3 (6.7)	186 (9.7)
*KRAS* G13D	3 (6.7)	167 (8.7)
*KRAS* Q61R	2 (4.4)	28 (1.5)
*KRAS* G12A	1 (2.2)	43 (2.2)
*KRAS* Q61L	1 (2.2)	10 (0.5)
*KRAS* A146T	1 (2.2)	51 (2.7)
*KRAS* A146V	1 (2.2)	14 (0.7)
*NRAS* G12D	1 (2.2)	19 (1.0)
Others	0 (0)	159 (8.3)
*BRAF*		
Wild-type	43 (95.6)	1,842 (96.2)
Mutated		
Class I	1 (2.2)	36 (1.9)
Class II	0 (0)	14 (0.7)
Class III	1 (2.2)	22 (1.1)
Unclassified	0 (0)	1 (0.1)

Abbreviation: AD, adenocarcinoma.

**TABLE A3.** Status of *ERBB3* in Anal AD

Genomic Alterations	Anal AD (n *=* 45), No. (%)
*ERBB3*	
Wild-type	35 (77.8)
Small-scale variant	
G284R (extracellular domain II)	5 (11.1)
V104L (extracellular domain I)	2 (4.4)
A232V (extracellular domain II)	1 (2.2)
R1118Q (C-terminal tail)	1 (2.2)
Amplification	1 (2.2)

Abbreviation: AD, adenocarcinoma.
